# Fastformer: An Efficient Attention-Based Framework for Rapid Multi-Class Fault Diagnosis in High-End Equipment Vibration Signals

**DOI:** 10.3390/e28070820

**Published:** 2026-07-19

**Authors:** Xiaohan Zhang, Hailun Dai, Chong Zhou, Qi Shen

**Affiliations:** 1 School of Finance, Southwestern University of Finance and Economics, Chengdu 611130, China; zhangxh@swufe.edu.cn (X.Z.); 124020204002@smail.swufe.edu.cn (C.Z.); 2 School of Business Administration, Southwestern University of Finance and Economics, Chengdu 611130, China; 1251202z9002@smail.swufe.edu.cn

**Keywords:** Fastformer, cross-entropy objective, Empirical Mode Decomposition, encoder-oriented attention, Margin-Enhanced Fault Softmax, multi-class fault diagnosis

## Abstract

Rapid and accurate multi-class fault diagnosis is essential for high-end equipment because different fault categories require different maintenance responses. This study aims to develop a lightweight and discriminative diagnostic framework that can identify multiple fault categories from non-stationary vibration signals while reducing redundant computation. High-frequency vibration signals provide direct condition information, but long sequences, noise, nonlinear dynamics, and non-stationary behavior make raw-signal classification unreliable. From an entropy-based information-processing perspective, the key issue is to separate informative fault modes from redundant fluctuations and enlarge inter-class distinctions in the probabilistic decision space. This study proposes Fastformer, an integrated framework for vibration-based fault identification. Empirical Mode Decomposition first converts each signal into Intrinsic Mode Functions to reduce modal mixing and preserve fault-related oscillatory components. The resulting components are processed by an encoder-oriented Q/K/V dot-product scoring mechanism, which constructs compact spatiotemporal embeddings without adopting a complete Transformer architecture. Validation-guided pruning removes low-contribution attention responses, while a Margin-Enhanced Fault Softmax classifier optimized with a cross-entropy-based objective strengthens category separation. By combining stable decomposition, lightweight attention scoring, pruning, and probabilistic margin learning, Fastformer achieves faster and more stable convergence. On the XJTU-SpurGear dataset, Fastformer obtains precision, recall, F1-score, and AUC values of 1.000. Additional validation on the HUST bearing dataset further shows that Fastformer achieves the best overall performance among the compared methods, with an AUC value of 0.9596.

## 1. Introduction

Safety-critical high-end equipment is widely deployed in advanced engineering systems, including aerospace propulsion, power-generation facilities, and emergency-response infrastructure [1,2,3]. In these settings, fault diagnosis must be both timely and specific, because different fault types may lead to different maintenance actions and risk-control strategies. Aero-engine equipment is a representative case. Its vibration signals contain rich information about operating states and component degradation, but they are usually affected by high dimensionality, noise, nonlinear dynamics, and non-stationary behavior. These properties make it difficult to distinguish fault-related patterns from background fluctuations. If early abnormal states are not recognized in time, minor degradation may develop into unplanned shutdowns, higher maintenance costs, or safety-related consequences [4,5].

High-frequency sensors installed on aero-engine equipment continuously record vibration responses during operation [6,7,8,9]. Although these measurements provide a direct basis for health assessment, a direct learning scheme based on raw sequences is not always reliable. Many diagnostic procedures implicitly assume that preprocessing alone can make the signal sufficiently stable for classification. In practice, however, operating-condition variation, missing fragments, local disturbances, and nonlinear mechanical responses may still remain in the processed data. Therefore, the collected vibration sequences need to be cleaned, filtered, completed, normalized, and segmented into fixed-length samples before representation learning [10,11,12]. This step reduces low-quality inputs and provides a more consistent basis for subsequent model training.

Empirical Mode Decomposition (EMD) is introduced to further organize the non-stationary signal into a structured time-frequency form [13,14,15]. The key reason for using EMD is that aero-engine faults are not always expressed in a single frequency band or a fixed temporal location. Instead, fault-related oscillations may appear across different scales and may overlap with normal dynamic responses. As an adaptive decomposition method, EMD does not depend on predefined basis functions and can separate the original signal into Intrinsic Mode Functions (IMFs) [16]. These IMF components provide a more stable representation of local oscillatory modes, helping to reduce modal overlap while preserving degradation-related information. After decomposition, Fastformer uses the IMF components as the input representation for relation modeling. The full Transformer architecture is not directly adopted, since complete attention structures may be unnecessarily heavy for long vibration sequences. Instead, Fastformer uses an encoder-oriented parameter organization process based on query, key, and value representations [17,18,19]. Through Q/K/V dot-product scoring, the model forms compact spatial embeddings of IMF components and compares temporal responses associated with different fault modes. This design allows fault-formation relationships to be explored without introducing the full complexity of a standard Transformer. Validation-guided pruning is further used to remove low-contribution responses and retain fault-sensitive dependencies [3,20].

The final diagnostic stage is designed for multi-class fault identification rather than simple abnormality detection. This distinction is important for industrial use, because recognizing the specific fault category can support more targeted maintenance decisions. The classifier therefore estimates the probability distribution among normal states and multiple fault severities. A margin-aware Softmax strategy is included to enhance category-level separability, so that similar fault patterns can be distinguished more clearly in the decision space.

Overall, Fastformer is designed as an integrated diagnostic framework for industrial vibration monitoring. EMD is used to obtain compact and more stable IMF representations, lightweight Q/K/V scoring is used to capture relationships among fault-sensitive components, and margin-aware classification is used to improve multi-class separability. This combination supports rapid fault-category recognition while keeping the computational process practical for high-frequency monitoring scenarios. The following sections present the process demonstration, algorithmic workflow, acceleration mechanism, parameter settings, benchmark comparisons, and experimental analysis.

## 2. Materials and Methods

### 2.1. Related Work

Existing studies on vibration-based fault diagnosis can be understood from two closely related objectives: obtaining a cleaner representation of non-stationary signals and learning discriminative relationships among different fault categories. Traditional feature extraction methods, including Principal Component Analysis (PCA), Independent Component Analysis (ICA), and Linear Discriminant Analysis (LDA), have been applied to reduce signal dimensionality and obtain representative features [21,22,23,24]. However, these methods usually transform the signal in a static manner and have limited ability to describe degradation information distributed across multiple temporal scales. When fault-related oscillations overlap with noise or when several fault modes appear in similar frequency ranges, the extracted features may not sufficiently separate the underlying modal components.

Deep learning methods have improved automatic feature learning for complex vibration signals [19,25]. CNN-based models are effective in capturing local waveform patterns, but they may miss long-range degradation trends because of their limited receptive fields. RNN-based models and Bidirectional Long Short-Term Memory (BiLSTM) networks can process sequential information, yet they may compress or lose key fault information when the input sequence is long and contains multiple oscillatory modes [26,27,28]. These limitations are particularly important in multi-class diagnosis, where different faults may share similar local responses but differ in their temporal distribution and physical degradation process.

Transformer-based models are effective in capturing global dependencies, but their complete architectures are often computationally heavy for long vibration sequences [19,29,30]. To keep the diagnostic model lightweight, Fastformer does not directly reproduce the full Transformer structure. Instead, it adopts the parameter organization process of the encoder module, where Q/K/V projections and dot-product scoring are used to construct compact spatial embeddings of IMF components. These embeddings allow the model to compare different temporal responses and identify the physical relationships underlying fault formation, such as shared oscillatory patterns, transient disturbances, and class-specific degradation trends. In this design, EMD first reduces modal overlap in the original non-stationary signal and produces more stable IMF components. The subsequent encoder-oriented scoring process then extracts fault-sensitive relations from these components with lower computational burden. Previous studies have often improved either signal preprocessing or model architecture separately [20,31,32]. Fastformer connects both purposes in one diagnostic pipeline: EMD is used to reduce modal mixing at the signal level, lightweight encoder-oriented embedding is used to capture fault-formation relationships, and a margin-aware classification strategy is introduced to improve the separation among fault categories.

Compared with Informer and Autoformer, which reduce complexity through ProbSparse self-attention with distilling and through series decomposition combined with auto-correlation, respectively, Fastformer keeps the standard Q/K/V dot-product formulation and lowers computation through a validation-guided pruning threshold instead [33]. The two lines of work also differ in purpose: Informer and Autoformer are developed for long-sequence forecasting, whereas Fastformer targets multi-class fault diagnosis. Accordingly, EMD is introduced here as a physically motivated decomposition for modal mixing in vibration signals, and the margin-enhanced Softmax is used to strengthen inter-class separability, neither of which is addressed by these forecasting-oriented lightweight attention models.

### 2.2. The Process Demonstration of Fastformer

**Figure 1:**
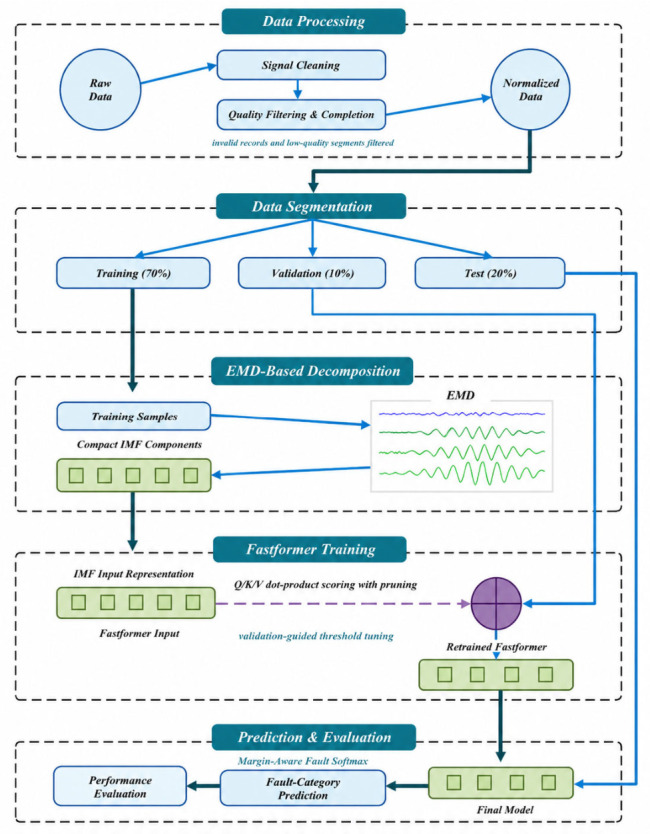
The whole workflow of Fastformer.

Figure 1 shows the overall workflow of the proposed Fastformer framework. High-frequency vibration signals are first collected from sensors and processed through signal cleaning, quality filtering, and data completion. Invalid records and low-quality segments are removed at this stage to reduce redundant inputs before model training. The normalized data are then divided into training, validation, and test subsets. The training samples are decomposed by Empirical Mode Decomposition (EMD), which produces compact Intrinsic Mode Function (IMF) components and represents the original non-stationary signal in a more structured time-frequency form.

The IMF representation is subsequently used as the input of the Fastformer module. Rather than directly copying a standard Transformer structure, the model adopts Q/K/V-based dot-product scoring to estimate temporal relationships among IMF components. Low-contribution attention responses are pruned under validation-guided threshold tuning, which reduces unnecessary computation while retaining fault-sensitive temporal information. The retrained Fastformer is then connected to a fault-category prediction stage. In this stage, a fault-semantic margin Softmax layer is used to separate different fault categories more explicitly, helping to preserve inter-class distinctions and reduce modal confusion among similar fault patterns [34]. Finally, the trained model is evaluated on the test set to assess diagnostic accuracy and computational efficiency. This workflow links lightweight data screening, adaptive signal decomposition, attention-based temporal scoring, and rapid multi-class fault decision within a unified diagnostic process.

### 2.3. Acceleration Mechanism of Fastformer

The Fastformer framework is designed to reduce signal complexity before temporal modeling and to strengthen fault-category separation during final diagnosis. Given a segmented vibration sample $x_{i}(t)$, Empirical Mode Decomposition (EMD) is first used to decompose the non-stationary signal into K Intrinsic Mode Functions (IMFs) and a residual term as Equation (1):

(1) $$x_{i}(t)=\sum_{k=1}^{K}\;IMF_{i,k}(t)+r_{i}(t)$$

where $IMF_{i,k}(t)$ denotes the *k*-th oscillatory component of sample $x_{i}(t)$, and $r_{i}(t)$ is the residual component. This decomposition is theoretically motivated by the modal-mixing problem common in non-stationary vibration signals, where a single frequency band may contain oscillatory components from multiple physical sources. By adaptively separating the signal into approximately mono-component IMFs, EMD brings each component closer to a locally stationary, narrow-band form, which provides a more favorable basis for the temporal relation modeling performed in the subsequent stage [35]. The selected IMF components are then organized into the compact input representation in Equation (2):

(2) $$H_{i}=\left[IMF_{i,1},IMF_{i,2},\ldots ,IMF_{i,K}\right]$$

For temporal relation modeling, the Q/K/V projections are constructed from the IMF representation, as defined in Equation (3):

(3) $$Q_{i}\;=\;H_{i}W_{Q},{\;K}_{i}\;={\;H}_{i}W_{K},\;\;V_{i}\;=\;H_{i}W_{V}$$

The dot-product relation score is calculated by Equation (4):

(4) $$S_{i}\;=\;\frac{Q_{i}K_{i}^{T}}{\sqrt{d_{k}}}$$

It is worth noting that the dot-product formulation itself (Equations (3) and (4)) follows the standard scaled dot-product attention design; its role here is to quantify pairwise similarity among IMF-derived temporal segments, which provides a principled basis for identifying fault-correlated dependencies once the input has been decomposed by EMD. The specific contribution therefore lies in the coupling between this scoring mechanism and the EMD-based input representation, rather than in the scoring formulation itself. A validation-guided 0–1 attention-pruning mask is introduced to determine whether each attention response is retained in Equation (5):

(5) $$M_{ij}=\left\{\begin{array}{c} 1,\;\;if\;S_{ij}>\tau \\ 0,\;\;otherwise\; \end{array}\right.$$

where τ is selected using the validation set. This mask is used only to remove low-contribution attention responses and does not define a binary diagnostic task. The pruned attention representation is computed according to Equation (6):

(6) $$A_{acc}(Q,K,V)=softmax(\tilde{S})V,{\tilde{\;S}}_{ij}=\left\{\begin{array}{cc} S_{ij}, & {\;M}_{ij}=1\\ -\infty , & {\;M}_{ij}=0 \end{array}\right.$$

The acceleration factor [36] is defined in Equation (7):

(7) $$\alpha =\frac{\;\mathrm{Operations}\;\mathrm{with}\;\mathrm{pruning}\;}{\;\mathrm{Operations}\;\mathrm{without}\;\mathrm{pruning}\;}$$

The corresponding speedup is given in Equation (8):

(8) $$S_{\mathrm{speed}\;}=\frac{1}{\alpha }$$

At the final diagnostic stage, a margin-enhanced multi-class Softmax is used to distinguish normal states and multiple fault severities. For sample i, the probability assigned to class c is defined in Equation (9):

(9) $$p_{i,c}=\frac{exp\left(z_{i,c}-I\left(c=y_{i}\right)m_{c}\right)}{\sum_{l=1}^{C}exp\left(z_{i,l}-I\left(l=y_{i}\right)m_{l}\right)}$$

where C is the number of diagnostic categories, $z_{i,c}$ is the logit of sample i for class c, $y_{i}$ is the true class label, $m_{c}\geq \;$0 is the class-level decision margin, and I(·) is the indicator function. From a theoretical standpoint, introducing a class-dependent margin enlarges the decision gap between the target class logit and competing classes in Equation (9), which corresponds to a wider separation between class regions in the logit space. Margin-based objectives of this form are commonly associated with improved inter-class separability and tighter generalization behavior compared with standard cross-entropy, which motivates the use of the margin-aware formulation for distinguishing fault categories with similar degradation signatures [37]. The margin term is applied to the target class during training, so the model is required to learn more separable representations for different fault categories.

The corresponding multi-class margin-aware cross-entropy objective [38] is written in Equation (10):

(10) $$L_{MAF}=-\frac{1}{N}\sum_{i=1}^{N}\sum_{c=1}^{C}I\left(c=y_{i}\right)logp_{i,c}$$

## 3. Results

### 3.1. Parameter Settings of the Model

Table 1 reports the main hyperparameter settings used in the Fastformer experiment. Since no single hyperparameter configuration can be assumed to be universally optimal for different diagnostic tasks, the main parameters of Fastformer were selected from predefined candidate ranges according to the validation behavior and the characteristics of the vibration signals [39]. The number of retained IMF components $\left(K\right)\;$ was selected from [2,3,4,5] and set to 3. A smaller value may discard useful fault-related oscillatory information, whereas a larger value may introduce redundant components and increase the burden of subsequent relation modeling. The number of encoder scoring layers $\left(L_{e}\right)\;$ and Q/K/V scoring heads $\left(h\right)\;$ were both set to 2. This setting provides sufficient capacity for constructing fault-sensitive temporal representations, while avoiding the unstable optimization and additional computation caused by deeper or wider attention scoring structures. The fault-class margin $\left(m_{c}\right)\;$ was chosen as 0.15 from [0.05, 0.10, 0.15, 0.20]. A smaller margin may not sufficiently enlarge the decision gap among similar fault categories, whereas an excessively large margin may make optimization more difficult. For model optimization, the learning rate was set to 0.001, the batch size was set to 32, and the number of training epochs was set to 100. These settings provided stable convergence in the tested experiments. Adam was adopted as the optimizer because it showed more stable parameter updating than the alternative optimizers considered. The attention threshold $\left(\tau \right)$ was selected as 0.20 from [0.10, 0.15, 0.20, 0.25, 0.30]. A lower threshold retains more low-contribution attention responses, while a higher threshold may remove useful fault-related dependencies. Therefore, τ=0.20  was used to balance informative relation preservation and redundant computation reduction.

### 3.2. The Introduction of the Dataset

The XJTU-Spur Gear dataset was used as the experimental benchmark to examine the proposed model under a multi-class vibration diagnosis setting. The dataset records gear vibration responses under different health conditions and contains crack-size labels that reflect progressive fault severity [40]. In this study, the original normal and fault samples were reorganized into a unified five-class diagnostic task. The label Anomaly was used to indicate the corresponding crack condition, including 0, 0.2, 0.6, 1.0, and 1.4 mm, where 0 represents the healthy state and the remaining labels denote different degrees of gear damage [41]. Since the signals were collected at 10,000 Hz, the dataset provides sufficient temporal detail for capturing weak fault responses and fine-grained degradation differences. To further examine the generalizability of Fastformer beyond the XJTU-Spur Gear dataset, the HUST bearing dataset was introduced as an additional validation dataset [42]. According to the dataset description, HUST contains vibration signals from bearings under 9 health states and 11 operating conditions, resulting in 99 raw Excel files. Each sampling file contains 262,144 data points, recorded at a sampling frequency of 25.6 kHz. In this study, the original health states were reorganized into a three-class diagnostic setting, including normal, minor fault, and severe fault.

In this study, all segmented samples were divided into training and test subsets at a ratio of 7:3. The training and test samples were kept strictly independent, and no test samples were used during model training, hyperparameter selection, or threshold determination, thereby avoiding data leakage between the training and evaluation stages. In addition, a repeated validation strategy was adopted instead of a full cross-validation procedure [43]. The experiment was repeated twice with different random splits, and the average results were reported as the final performance to reduce the influence of random partitioning. The main dataset information used in the experiment is summarized in Table 2.

### 3.3. The Performance Evaluation

Model performance was evaluated using the confusion matrix, which provides a class-wise summary of the relationship between predicted labels and true labels. Since the present task involves five diagnostic categories, the basic counts are calculated for each class by treating the target class as positive and all remaining classes as non-target categories. The four derived quantities are defined as follows:

- True Positive ($T_{p}$): the number of samples from a given class that are correctly assigned to that class.
- False Positive ($F_{p}$): the number of samples from other classes that are incorrectly predicted as the target class.
- True Negative ($T_{n}$): the number of samples that neither belong to the target class nor are predicted as that class.
- False Negative ($F_{n}$): the number of samples from the target class that are incorrectly classified into other categories.

These class-wise counts are then used to calculate the main performance indicators described below.

1. Precision: evaluates the proportion of samples predicted as a specific fault category that are correctly assigned to that category, as defined in Equation (11).

(11) $$Precision=T_{P}/\left(T_{P}+F_{P}\right)$$

2. Recall: measures the proportion of samples from a specific fault category that are successfully identified by the model, as defined in Equation (12).

(12) $$Recall=T_{P}/\left(T_{P}+F_{N}\right)$$

3. $F_{1}$ represents the harmonic balance between precision and recall, as defined in Equation (13).

(13) $$F_{1}\;score=2*\left(Precision*Recall\right)/\left(Precision+Recall\right)$$

4. AUC assesses the overall class-discrimination capability of the model under different decision thresholds. The true positive rate and false positive rate are first calculated according to Equation (14).

(14) $$TPR=\frac{T_{P}}{T_{P}+F_{N}},\;\;FPR=\frac{F_{P}}{F_{P}+T_{N}}$$

Then the AUC is obtained as the area under the ROC curve, as defined in Equation (15).

(15) $$AUC=\int_{0}^{1}TPR(FPR)d(FPR)$$

### 3.4. The Benchmark Methods Setting

To evaluate the diagnostic performance of Fastformer, several representative benchmark methods were selected for comparison. These methods cover conventional machine-learning models, deep-learning sequence models, hybrid diagnostic models, and attention-based architectures. GMM and SVM were used as traditional machine-learning baselines, while EMD-BiLSTM and GMM-BiLSTM were included to examine the effect of combining signal processing or probabilistic modeling with recurrent deep learning. The standard Transformer was selected as a complete attention-based reference model. Fastformer was further compared with these methods to verify whether the proposed EMD-assisted and encoder-oriented attention framework can improve multi-class fault recognition while reducing redundant computation. To ensure a fair comparison, the hyperparameters of all benchmark models were selected under the same validation-based principle as Fastformer, rather than directly using arbitrary default settings. For each benchmark, the final configuration was determined according to its validation performance and training stability under the same training-test partition. The methodological categories of the compared models are summarized in Table 3.

### 3.5. Experiment Results

The diagnostic results of all benchmark methods are summarized in Table 4. The table reports the class-wise precision, recall, F1-score, support, and overall AUC-score for each model under the five-class fault diagnosis setting. These metrics provide a direct comparison of the ability of different methods to distinguish normal conditions and multiple fault severities. Based on these results, the following analysis further discusses the classification performance, class-level stability, and overall discriminative capability of the proposed Fastformer framework.

According to Table 4, the compared methods show clear differences in five-class fault recognition. GMM obtains an AUC-score of 0.3169, and its precision, recall, and F1-score remain close to zero for several fault categories, indicating that a simple probabilistic structure is not sufficient for separating complex vibration fault patterns. SVM performs better, with an AUC-score of 0.9859, but its class-wise performance still shows slight variation across different fault severities. EMD-BiLSTM achieves strong results, with an AUC-score of 0.9992, which suggests that decomposing vibration signals before sequence learning can improve the representation of fault-related information.

Compared with these benchmark methods, Fastformer achieves precision, recall, F1-score, and AUC-score values of 1.0000 under the five-class setting. This result indicates that the proposed framework can distinguish normal samples and multiple fault severities with high stability in the current experimental setting. The performance is consistent with the design logic of Fastformer: EMD reduces modal mixing in non-stationary vibration signals and decomposes noise-affected industrial vibration data into more structured modal components; the encoder-oriented Q/K/V scoring process then builds compact spatiotemporal representations from these components, and the margin-enhanced Softmax objective further enlarges the decision gap among different fault categories. In contrast, the standard Transformer obtains an AUC-score of 0.8527. This lower performance may be attributed to its direct use of raw or weakly decomposed vibration sequences, where noise, modal overlap, and local non-stationary fluctuations make attention learning less stable. Without EMD-based modal organization and margin-enhanced class separation, the complete attention structure may capture redundant temporal relations rather than fault-sensitive patterns, making it less suitable for this task.

To further examine the reliability of the reported results, the test predictions of Fastformer were checked after model evaluation. Under the current experimental setting, no misclassified samples were observed in the test subset. Therefore, a conventional error analysis based on wrongly classified cases could not be conducted. This result may be related to the controlled laboratory environment of the XJTU-Spur Gear dataset, the clear crack-size labels, and the strong separability of the decomposed vibration patterns. In addition, the experiment was repeated with different random training-test partitions, and the average performance was used to reduce the influence of a single data split [44]. These results suggest that Fastformer performs stably under the present dataset, but they should still be interpreted within the current controlled experimental condition rather than directly generalized to all industrial scenarios.

To further examine the generalizability of Fastformer under the same type of vibration-based fault diagnosis but on a different equipment platform, an additional validation was conducted on the HUST bearing dataset. Both XJTU-Spur Gear and HUST are laboratory-controlled rotating machinery vibration datasets, and both contain fault-related oscillatory patterns that can be used for health-state identification. However, they differ in equipment object and fault organization: XJTU focuses on gear crack-size degradation, whereas HUST focuses on bearing health states under multiple operating conditions. Therefore, HUST provides a complementary validation scenario for examining whether the proposed framework can maintain its diagnostic advantage beyond the original gear dataset. As shown in Table 5, Fastformer achieves the best overall performance on HUST, with an AUC-score of 0.9596. Although this performance is lower than that on XJTU, Fastformer still outperforms GMM, SVM, EMD-BiLSTM, GMM-BiLSTM, and the standard Transformer. This result suggests that the proposed framework is not limited to a single dataset and can retain stable diagnostic capability across different rotating machinery vibration datasets.

Overall, the results support the effectiveness of combining adaptive signal decomposition, lightweight attention scoring, and multi-class probabilistic optimization for rapid fault diagnosis in high-end equipment.

### 3.6. Ablation Analysis of Convergence Behavior

**Figure 2:**
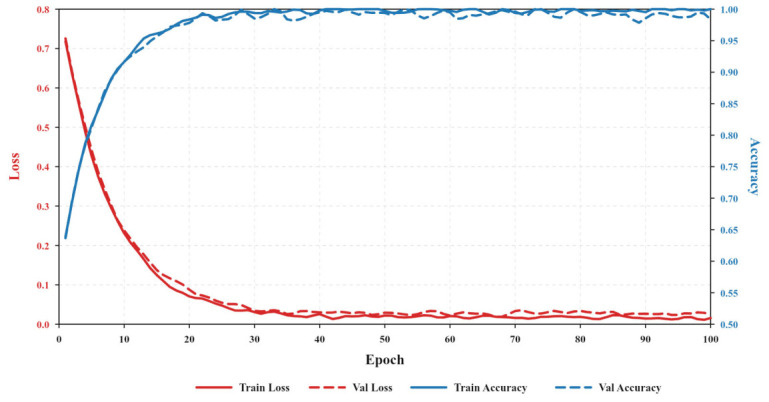
Convergence curves of Fastformer during training and validation.

**Figure 3:**
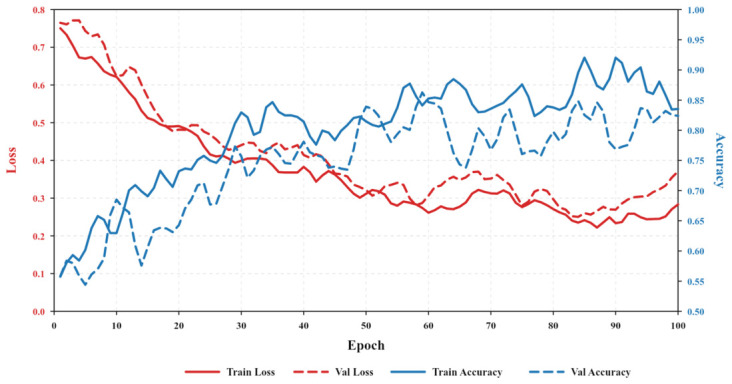
Convergence curves of the standard Transformer during training and validation.

**Figure 4:**
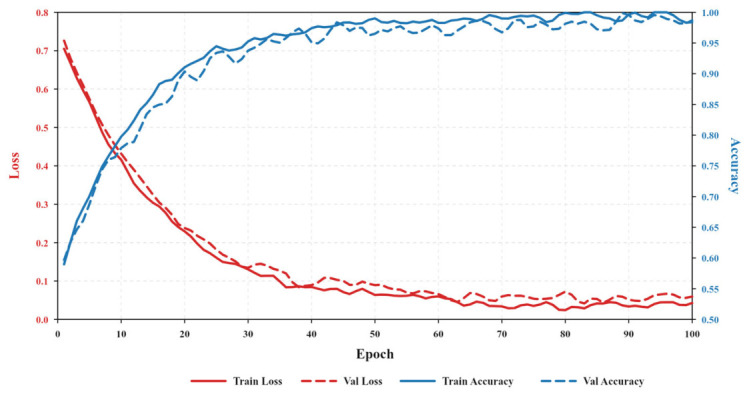
Convergence curves of EMD-BiLSTM during training and validation.

To further evaluate the contribution of the proposed structure, an ablation analysis was carried out by comparing the convergence curves of Fastformer, the standard Transformer, and EMD-BiLSTM, as shown in Figure 2, Figure 3 and Figure 4. The comparison records the training loss, validation loss, training accuracy, and validation accuracy over 100 epochs. Because model optimization is based on mini-batch learning, small fluctuations appear during training and validation. These variations are expected in vibration-signal classification, where samples contain noise, non-stationary changes, and class-level differences. Therefore, the convergence trend, rather than point-wise fluctuation, is used to assess the training stability and learning efficiency of each model.

Figure 2 shows that Fastformer reduces loss quickly in the early epochs and reaches a stable high-accuracy region within a short training period. This result suggests that EMD first provides clearer IMF components by reducing modal mixing, while the encoder-oriented Q/K/V scoring mechanism can efficiently organize these components into fault-sensitive representations. Figure 3 indicates that the standard Transformer converges more slowly and exhibits larger accuracy fluctuations. This behavior implies that directly applying a complete attention architecture may introduce redundant representation learning and unstable optimization for vibration sequences. Figure 4 shows that EMD-BiLSTM benefits from decomposition-based preprocessing and produces smoother convergence than the standard Transformer, but its recurrent structure still requires more epochs to approach a stable state. Notably, this comparison also offers an indirect, component-level perspective on the proposed design. EMD-BiLSTM retains the same EMD-based decomposition as Fastformer, but its recurrent structure propagates information through sequential neuron states, which limits its capacity to capture medium- and long-range temporal dependencies; this is reflected in its slightly lower AUC-score of 0.9992. The standard Transformer, in contrast, retains a complete attention mechanism capable of longer-range dependency modeling but omits signal-level decomposition, and its AUC-score drops markedly to 0.8527. The fact that Fastformer, which combines both modal decomposition and encoder-oriented attention scoring, outperforms both baselines therefore provides empirical support for the individual contributions of these two components, complementing the theoretical justification given in Section 2.3. In addition, Fastformer shows a faster decrease in training and validation loss than the standard Transformer, suggesting that the proposed lightweight organization improves the efficiency and stability of gradient-based optimization.

Overall, the ablation results show that Fastformer achieves faster and more stable convergence than the two reference models. This finding supports the design logic of the proposed framework: EMD improves the quality of signal representation, encoder-oriented attention scoring accelerates temporal relation learning, and the resulting structure is more suitable for rapid multi-class fault diagnosis in high-end equipment vibration signals.

### 3.7. Parameter Sensitivity Analysis of Attention Threshold and Retained IMF Components

**Figure 5:**
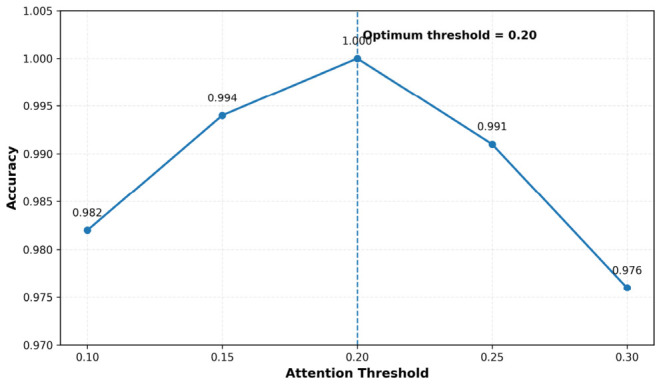
Sensitivity of diagnostic accuracy to attention threshold τ on the XJTU-Spur Gear dataset.

To examine the robustness of the key parameter settings in Fastformer, sensitivity analyses were conducted on the XJTU Dataset for the attention pruning threshold τ and the number of retained IMF components K. For the attention threshold, five candidate values, 0.10, 0.15, 0.20, 0.25, and 0.30, were evaluated. As shown in Figure 5, the diagnostic accuracy first increases and then decreases with the change in τ. A relatively small threshold retains more attention responses, but may also preserve low-contribution dependencies and weaken the compactness of the learned representation. When τ increases to 0.20, redundant responses are effectively reduced while fault-sensitive temporal relations are still preserved, leading to the highest accuracy of 1.000. Further increasing the threshold results in performance degradation, suggesting that excessive pruning may remove informative fault-related dependencies.

**Figure 6:**
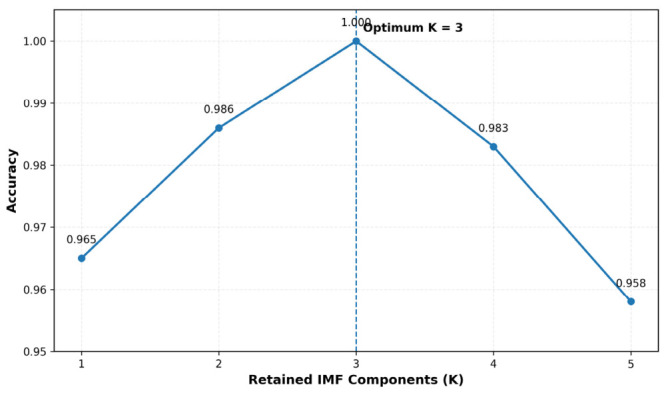
Sensitivity of diagnostic accuracy to retained IMF components K on the XJTU-Spur Gear dataset.

The sensitivity of K shows a comparable trend. As shown in Figure 6, the diagnostic accuracy reaches its highest value when three IMF components are retained. When K is insufficient, the decomposed representation may fail to capture adequate oscillatory modes associated with fault evolution. Conversely, retaining too many IMF components may introduce weakly informative or redundant modes, which increases the complexity of subsequent attention scoring without providing proportional diagnostic gains. Therefore, K=3  provides a suitable balance between modal information preservation and redundant component suppression. Based on these observations, τ=0.20  and K=3  were adopted in the final Fastformer configuration. The two sensitivity analyses jointly indicate that the performance of Fastformer depends on a controlled balance between information retention and structural compactness, which is consistent with the lightweight design principle of the proposed framework.

### 3.8. Computational Entropy Analysis

To further evaluate the computational organization of different diagnostic models, this study introduces computational entropy to describe how the computational burden is distributed across major processing modules. This indicator focuses on the structural allocation of computation, namely whether the cost is concentrated in a dominant module or distributed across several functional stages [45]. For a diagnostic model with m computational sub-processes, the normalized computational proportion of the i-th sub-process is defined as Equation (16):

(16) $$p_{i}=\frac{C_{i}}{\sum_{j=1}^{m}C_{j}}$$

where $C_{i}$ denotes the estimated computational cost of the i-th sub-process. Based on this definition, the computational entropy of a model is calculated as Equation (17):

(17) $$H_{c}=\;-\;\sum_{i=1}^{m}p_{i}logp_{i}$$

As reported in Table 6, the compared methods show different computational-entropy characteristics. GMM, SVM, GRU, and BiLSTM have relatively low entropy values, indicating that their computation is mainly concentrated in a small number of modeling or recurrent updating operations. EMD-BiLSTM and GMM-BiLSTM show higher entropy values because decomposition or probabilistic modeling is introduced before sequence learning. The standard Transformer reaches the highest entropy value, suggesting that its computation is distributed across embedding, attention, feed-forward transformation, and classification modules. Fastformer records an intermediate value of 1.12. This result indicates that the proposed model retains necessary functional modules, including EMD, Q/K/V relation scoring, attention pruning, and margin-enhanced classification, while avoiding the broader computational dispersion of the full Transformer structure. Thus, Fastformer improves fault-sensitive representation with a function-oriented lightweight design rather than by simply stacking additional modules.

### 3.9. Numerical Ablation Analysis of Key Modules

**Figure 7:**
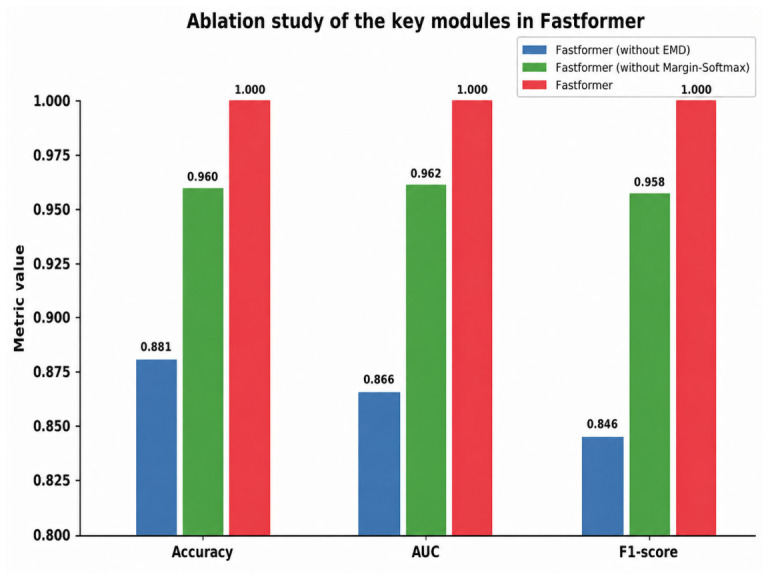
Numerical ablation results of the key modules on the XJTU-Spur Gear dataset.

To quantitatively evaluate the contributions of the key modules, an ablation study was conducted by separately removing EMD and Margin-Softmax from Fastformer. As shown in Figure 7, the complete Fastformer achieves Accuracy, AUC, and F1-score values of 1.000. After removing EMD, these metrics decrease to 0.881, 0.866, and 0.846, respectively, indicating that EMD plays a major role in reducing modal mixing and preserving fault-sensitive oscillatory information. When Margin-Softmax is removed, the corresponding values decrease to 0.960, 0.962, and 0.958, showing that the margin-based classifier further improves inter-class separability, although its effect is smaller than that of EMD. Overall, the ablation results confirm that both modules contribute to the final diagnostic performance, with EMD providing the primary representation improvement and Margin-Softmax further strengthening fault-category discrimination.

## 4. Discussion

The experimental results show that Fastformer performs more consistently than the compared machine-learning and deep-learning baselines in the five-class vibration diagnosis task. Compared with GMM and SVM, the proposed framework does not rely only on static decision boundaries or shallow statistical separation, which are often insufficient for nonlinear and non-stationary vibration signals. Compared with recurrent models such as EMD-BiLSTM and GMM-BiLSTM, Fastformer avoids excessive dependence on sequential memory propagation and instead uses encoder-oriented Q/K/V scoring to construct compact spatiotemporal representations. The difference between Fastformer and the standard Transformer is also meaningful. A complete attention architecture can model long-range dependencies, but it may introduce redundant computation and unstable optimization when directly applied to vibration sequences. Fastformer keeps the relation-scoring advantage of the encoder structure while using validation-guided pruning to remove low-contribution attention responses. This explains why the model achieves high diagnostic accuracy and faster convergence in the current experiment.

From a broader perspective, the results suggest that the effectiveness of Fastformer comes from the cooperation of three modules rather than from a single classifier. EMD reduces modal mixing and converts raw vibration data into more interpretable IMF components, which improves the quality of the input representation. The lightweight encoder-oriented attention mechanism then captures temporal relations among fault-sensitive components without copying the full Transformer structure. Finally, the margin-enhanced Softmax objective strengthens the decision boundary among different fault categories, which is important for multi-class diagnosis where similar degradation levels may be confused. This modular logic may also be useful for other high-end equipment monitoring problems involving noisy, long, and non-stationary signals. Future studies can further test the framework on more datasets, cross-load operating conditions, imbalanced fault samples, and online monitoring scenarios to verify its robustness and industrial applicability.

## 5. Conclusions

This study proposed Fastformer as an integrated framework for rapid and accurate multi-class fault diagnosis in high-end equipment vibration signals. The framework is organized around a coherent diagnostic chain. First, Empirical Mode Decomposition provides stable signal decomposition by converting non-stationary vibration sequences into IMF components, which reduces modal mixing and preserves fault-related oscillatory information. Second, the encoder-oriented Q/K/V scoring process constructs compact spatiotemporal representations and captures relationships among fault-sensitive components without adopting a complete Transformer structure. Third, validation-guided attention pruning removes low-contribution responses and reduces redundant computation. Finally, the Margin-Enhanced Fault Softmax classifier, optimized through a cross-entropy-based objective, enlarges inter-class decision differences and improves the separability of normal states and multiple fault severities.

The experimental results on the XJTU-SpurGear dataset verify the effectiveness of this linked design. Fastformer achieves precision, recall, F1-score, and AUC values of 1.000 under the five-class setting, while the attention pruning strategy reduces computational overhead by approximately 35–40%. These findings indicate that the proposed framework can improve diagnostic reliability and computational efficiency at the same time. Compared with traditional machine-learning methods, recurrent deep-learning models, and the standard Transformer, Fastformer shows stronger class-level stability and faster convergence, which supports its potential for vibration-based health monitoring in safety-critical industrial scenarios.

Future research should further examine the generalization and interpretability of Fastformer [46]. The framework can be tested under variable speeds, changing loads, cross-device conditions, imbalanced fault samples, and noisier industrial environments. In addition, interpretable visualization of IMF components, attention scores, pruning decisions, and class-margin effects can help explain how the model reaches each diagnostic decision. These extensions will be important for moving Fastformer from controlled experimental validation toward broader industrial deployment.

## Figures and Tables

**Table 1:** The Hyperparameters table.

Hyperparameters	Range	Value
Retained IMF Components (K)	[2, 3, 4, 5]	3
Encoder Scoring Layers ($L_{e}$)	[1, 2, 3]	2
Q/K/V Scoring Heads (h)	[2, 4, 8]	2
Fault-Class Margin ($m_{c}$)	[0.05, 0.10, 0.15, 0.20]	0.15
Learning rate	[0.001, 0.01, 0.1]	0.001
Batch size	[32, 64, 128]	32
Training Epochs	[50, 100, 150, 200]	100
Optimizer Options	[Adam, AdamW, RMSProp]	Adam
Attention Threshold (τ)	[0.10, 0.15, 0.20, 0.25, 0.30]	0.20

**Table 2:** Summary of the Experimental Datasets..

Dataset	Classification Setting	Data Environment
XJTU-Spur Gear Dataset	Five-class gear fault diagnosis based on crack-size labels: 0, 0.2, 0.6, 1.0, and 1.4 mm. The label 0 denotes the normal state, while the other four labels represent increasing fault severities.	Controlled laboratory measurements with experimentally induced fault patterns under stable operating conditions (Xi’an Jiaotong University platform); signals are collected in a stable and repeatable setting.
HUST Bearing Dataset	Three-class bearing fault diagnosis based on reorganized health states: normal, minor fault, and severe fault. The original 9 health states were grouped into Class 0, Class 1, and Class 2 according to health condition and fault severity. After fixed-length segmentation, the generated samples were divided into training and test subsets at a ratio of 7:3.	Controlled laboratory measurements from the HUST bearing platform. The dataset contains 9 health states and 11 operating conditions, resulting in 99 raw Excel files. Each file contains 262,144 data points collected at 25.6 kHz, supporting additional validation under different bearing health states and operating conditions.

**Table 3:** Classification of the Benchmark Methods.

Method	ML-Based	DL-Based	Attention-Based
GMM	√		
SVM	√		
EMD-BiLSTM		√	
GMM-BiLSTM	√	√	
Transformer		√	√
Fastformer		√	√

**Table 4:** Summary of Benchmark Setting Experimental Results in XJTU Dataset.

Model	Class	Precision	Recall	F1-Score	Support	AUC-Score
GMM	0	0.5026	0.8878	0.6418	9262	0.3169
	1	0.0000	0.0000	0.0000	10,094	
	2	0.0000	0.0000	0.0000	9423	
	3	1.0000	0.7790	0.8758	8452	
	4	0.0000	0.0000	0.000	9595	
SVM	0	1.0000	0.9909	0.9954	9262	0.9859
	1	0.9376	0.9999	0.9677	10,094	
	2	1.0000	0.9987	0.9994	9423	
	3	1.0000	0.9601	0.9797	8452	
	4	0.9946	0.9691	0.9817	9595	
EMD-BiLSTM	0	0.9993	0.9995	0.9994	9168	0.9992
	1	0.9992	0.9989	0.9992	10,140	
	2	0.9994	0.9987	0.9986	9504	
	3	0.9986	0.9996	0.9995	8450	
	4	0.9994	0.9995	0.9994	9263	
Fastformer	0	1.0000	1.0000	1.0000	9234	1.0000
	1	1.0000	1.0000	1.0000	10,143	
	2	1.0000	1.0000	1.0000	9472	
	3	1.0000	1.0000	1.0000	8469	
	4	1.0000	1.0000	1.0000	9200	
GMM-BiLSTM	0	0.9234	0.9264	0.9249	9340	0.9509
	1	0.9530	0.9552	0.9541	10,030	
	2	0.9820	0.9832	0.9826	9509	
	3	0.9709	0.9639	0.9674	8366	
	4	0.9259	0.9254	0.9257	9280	
Transformer	0	0.7773	0.8581	0.8157	9263	0.8527
	1	0.7325	0.8841	0.8012	9996	
	2	0.9651	0.9735	0.9693	9481	
	3	0.9554	0.7467	0.8382	8463	
	4	0.8769	0.7578	0.8130	9317	

**Table 5:** Summary of Benchmark Setting Experimental Results in HUST Dataset.

Model	Class	Precision	Recall	F1-Score	Support	AUC-Score
GMM	0	0.6187	0.7584	0.6812	426	0.5436
	1	0.4362	0.3658	0.3979	1683	
	2	0.4815	0.4173	0.4471	1696	
SVM	0	0.8976	0.8841	0.8908	419	0.8234
	1	0.6438	0.6075	0.6251	1698	
	2	0.7042	0.6636	0.6833	1686	
EMD-BiLSTM	0	0.9427	0.9516	0.9471	430	0.9348
	1	0.8235	0.8079	0.8156	1685	
	2	0.9128	0.9034	0.9081	1693	
Fastformer	0	0.9736	0.9812	0.9774	424	0.9596
	1	0.9048	0.8875	0.8961	1694	
	2	0.9627	0.9714	0.9670	1688	
GMM-BiLSTM	0	0.9032	0.9127	0.9079	417	0.9042
	1	0.7834	0.7568	0.7699	1697	
	2	0.8625	0.8461	0.8542	1684	
Transformer	0	0.7819	0.8426	0.8111	428	0.8531
	1	0.7164	0.8189	0.7642	1682	
	2	0.8793	0.7637	0.8174	1699	

**Table 6:** Computational entropy comparison of different models.

Method	Entropy Calculation (Normalized)	$H_{\mathrm{total}\;}$
GRU	Recurrent state update ≈0.64, Output projection ≈0.36	0.65
BiLSTM	Bidirectional recurrent update ≈0.69, Output projection ≈0.31	0.67
GMM	Gaussian mixture modeling ≈0.74, Class assignment ≈0.26	0.57
SVM	Kernel mapping ≈0.68, Decision boundary calculation ≈0.32	0.63
EMD-BiLSTM	EMD ≈0.22, BiLSTM sequence learning ≈0.60, Classifier ≈0.18	0.94
GMM-BiLSTM	GMM modeling ≈0.24, BiLSTM sequence learning ≈0.58, Classifier ≈0.18	0.97
Transformer	Embedding ≈0.14, Multi-head attention ≈0.43, Feed-forward transformation ≈0.29, Classifier ≈0.14	1.28
Fastformer	EMD ≈0.16,Q/K/V relation scoring ≈0.57, Attention pruning ≈0.09, Margin-enhanced classifier ≈0.18	1.12

## Data Availability

The data supporting this study are available upon reasonable request. The XJTU-Spur Gear and HUST bearing datasets used in this research are publicly accessible for academic purposes, with comprehensive details provided in the cited articles to promote transparency and ensure the reproducibility of the findings.
